# Clinical Grade Regulatory CD4^+^ T Cells (Tregs): Moving Toward Cellular-Based Immunomodulatory Therapies

**DOI:** 10.3389/fimmu.2018.00252

**Published:** 2018-02-13

**Authors:** Richard Duggleby, Robert David Danby, J. Alejandro Madrigal, Aurore Saudemont

**Affiliations:** ^1^Anthony Nolan Research Institute, London, United Kingdom; ^2^University College London, London, United Kingdom; ^3^Oxford University Hospitals NHS Foundation Trust, Oxford, United Kingdom

**Keywords:** regulatory T cells, suppression, immunotherapy, transplant, rejection, graft-versus-host disease

## Abstract

Regulatory T cells (Tregs) are CD4^+^ T cells that are key players of immune tolerance. They are powerful suppressor cells, able to impact the function of numerous immune cells, including key effectors of inflammation such as effector T cells. For this reason, Tregs are an ideal candidate for the development of cell therapy approaches to modulate immune responses. Treg therapy has shown promising results so far, providing key knowledge on the conditions in which these cells can provide protection and demonstrating that they could be an alternative to current pharmacological immunosuppressive therapies. However, a more comprehensive understanding of their characteristics, isolation, activation, and expansion is needed to be able design cost effective therapies. Here, we review the practicalities of making Tregs a viable cell therapy, in particular, discussing the challenges faced in isolating and manufacturing Tregs and defining what are the most appropriate applications for this new therapy.

## Introduction

One of the major challenges for allogeneic hematopoietic cell (HC) and solid organ transplantation is the continued interaction between donor and recipient immunity, requiring immunosuppressive therapy to prevent rejection and/or graft-versus-host disease (GvHD). Unfortunately, many standard immunosuppressive drugs cause global immunosuppression, impairing the beneficial immune response to infections and tumor surveillance. In addition, many pharmacological agents also have untoward side effects, including steroid induced diabetes, osteoporosis and proximal myopathy, causing morbidity and mortality (1). Using novel cellular therapies, such as regulatory T cells (Tregs), to provide suppressive function could provide an alternative solution to conventional pharmacological agents.

Regulatory T cells are a subset of T cells that act a key regulators of immune tolerance and essential for maintenance of immune homeostasis. Tregs are typically characterized as CD4^+^CD25^+^CD127^low^Foxp3^+^ T cells, although a subset of CD8^+^ regulatory T cells have also been reported in mice and humans but mainly in autoimmunity (2–6). CD8^+^ Tregs are less well characterized and therefore will not be discussed further in this review. Tregs exert their suppressive function using a variety of cell contact dependent and independent mechanisms (7, 8); Tregs express high levels of cytotoxic T-lymphocyte-associated protein 4 (CTLA-4) (9) and inhibit proliferation of T cells and B cells *via* this pathway in mice and humans (10, 11). In addition, CTLA-4 is involved in Treg-mediated suppression of dendritic cells (DCs) by causing up-regulation of indoleamine 2,3-dioxygenase (IDO) secretion in DC. In mainly animal models, this depletes local tryptophan, inducing apoptosis in T cells and inducing a regulatory DC phenotype (12–14). Tregs also have high expression of the high affinity IL-2 receptor (CD25, CD122, and C132), sequestrating IL-2 and inhibiting IL-2-dependent activation and proliferation of conventional T cells (8, 15) and, in mice NK cells (16, 17). Tregs bind TGF-β to their surface, with evidence that it mediates T cell (18) (murine studies), and NK cell suppression (19) (human studies), inducing IDO in DCs (14) (murine and human), and provide a positive feedback loop in which TGF-β induces and maintains FOXP3^+^ Tregs (20) (mouse). Murine studies also show that Tregs expressing soluble factors including IL-10 and IL-35 can confer suppressive function to other cell types, such as conventional T cells (infectious tolerance) (8, 21, 22). Finally, animal studies also indicate Tregs have cytotoxic T cell effects (23) and a number of indirect suppressive mechanisms, such as inhibition of antigen presentation (24), breakdown of extracellular ATP (a proinflammatory mediator) (25, 26) and metabolic disruption of target effectors (27). The relative importance and contribution of each mechanism *in vivo* remains uncertain. However, it has been clearly shown, in animal and human studies, that Tregs can inhibit the functions of multiple cell types including effector T cells, CD4 and CD8 T cells (28, 29), B cells (11), NKT cells (30), NK cells (19), DC (12, 31), monocytes, and macrophages (32).

In contrast to pharmacological agents, Treg-mediated immune suppression has the potential for specificity and allow the establishment of tolerance; with improvements in our knowledge of trafficking, it maybe possible to direct Tregs to specific tissues to achieve a level of local rather than systemic suppression. Allograft rejection animal models (33, 34) have shown that Tregs can prevent rejection through linked suppression. This is a form of bystander suppression, where tolerated and third-party antigens are presented by the same antigen-presenting cell (APC) or are present in the same tissue; such that Tregs become activated and suppress third-party antigen responses in addition to those of their cognate antigen (33). In these models, the grafts became tolerant through the generation and infiltration of Tregs into the tissues, conferring a form of immune privilege (33–35). Tregs, therefore, confer tolerance through “infectious tolerance” (35). As these concepts were developed in allograft rejection models, their relevance to the field of solid organ transplantation is clear (33, 34), establishing long-term tolerance to solid organ transplants.

When used in the context of allogeneic HC transplantation (HCT), Tregs may provide adequate immunosuppression to allow tolerance mechanisms to prevent GvHD and graft rejection. Initial observations supporting this hypothesis were established in early animal models of acute GvHD using irradiated recipient mice infused with allogeneic donor bone marrow (BM) and T cells, or non-irradiated SCID mice infused with allogeneic donor T cells. Using these models, Taylor et al. demonstrated that depletion of the Treg population from allogeneic donor CD4^+^ cells exacerbated the onset of GvHD, while the addition of polyclonal expanded Tregs (anti-CD3) inhibited GvHD (36). Similarly, Hoffmann et al. showed that donor Tregs isolated from splenocytes or BM can suppress acute GvHD caused by the addition of donor allogeneic BM and T cells to irradiated recipient mice (37). Extending this work, Edinger et al. showed, in a murine model with an A20 leukemia cell line, that donor BM alone could not control tumor growth. Addition of conventional T cells controlled the tumor but the mice died from acute GvHD. However, addition of conventional T cells and Tregs maintained the graft-versus-tumor response but prevented GvHD (38). At the same time, Cohen at al. showed in a similar animal model of GvHD, that donor Tregs expanded with recipient splenocytes could also control GvHD (39). Trenado et al., expanding with recipient allogenic APC, showed specific Tregs had an advantage over polyclonal Tregs in controlling experimental GvHD (40). More recently, human Tregs isolated under Good Manufacturing Protocol (GMP) compliant conditions were tested in a xenograft GvHD murine model (NSG mice with human CD3^+^ cells responding to human allogeneic DCs). In this model, both polyclonal and allogeneic DC expanded Tregs were able to improve GvHD (41). These animal studies, therefore, demonstrated that freshly isolated and/or expanded Tregs (polyclonal and allospecific) can reduce acute GvHD. Hence, these animal data provided the initial rationale for the investigation of Treg cellular therapies in human allogeneic HCT. In support, subsequent retrospective observational studies in human HCT have shown that acute GVHD is inversely associated with the frequency of Tregs (42). Moreover, data from analysis of Treg content in the HCT grafts demonstrated that the presence of higher proportions of Tregs was also beneficial for overall survival post-HCT (43).

While Treg cellular therapies may become an important therapeutic option, the possibility of infectious tolerance and bystander suppression has raised concerns regarding inhibition of normal antitumor and antimicrobial activity. As will be discussed within this review, clinical trials (to date) have not shown an increase in relapse and only one study reported a trend toward increased infections. This was not replicated in later studies in which higher Treg numbers were infused (44, 45).

In this review, we describe the current status of Tregs therapies and discuss the challenges that remain in making Tregs therapy practical, including cell dose requirement, methods of isolation and manufacture and specificity requirements.

## Producing Tregs for Clinical Use

### Source of Tregs

Regulatory T cells for clinical therapies can be obtained from peripheral blood (PB) or umbilical cord blood (UCB). Within both sources, CD4^+^CD25^+^CD127^low^FOXP3^+^ Tregs constitute approximately 5–10% of CD4^+^ cells, with CB Tregs being a more distinct population as they lack high numbers of CD25^int^CD127^hi^ cells (46, 47). While PB Tregs are predominately CD45RA^−^ (approximately 70%) (46, 48), the majority (>80%) of CB Tregs and CD4^+^ cells express the naive CD45RA^+^ phenotype (47, 48). CB Tregs are also CD31^+^, suggesting that they are direct thymic emigrants (49).

Although the overall proportions of Tregs are similar in PB and CB, there are conflicting reports as to whether the phenotypic differences translate into functional differences. Some studies have reported that CB Tregs do not have suppressive function (48, 50), while others find no differences between PB and CB (47). Although the source of this discrepancy is unclear, it may in part be due to the nature and timing of the *in vitro* functional assays being used. For example, Fujimaki et al. used anti-CD3/28 beads (48) to stimulate target (conventional effector T cells) and used Treg populations (autologous) from CB and found poor suppression compared to PB Tregs. Similarly, Thornton et al. measured suppression using a mixed lymphocyte reaction and found no suppression immediately postisolation (50). However, they did observe suppressive function by CB Tregs after 5-day culture with ovalbumin (50). By comparison, Santner-Nanan et al. used soluble anti-CD3 in the presence of allogenic APCs and found no difference in function between CB Tregs and PB Tregs from different age groups (47). Taken together this would suggest that unlike PB Tregs, CB Tregs require specific stimuli (or maturation) to be able to demonstrate suppressive function.

The predominance of naive CD45RA^+^ Tregs in CB could also have functional implications for Treg cellular therapy products obtained from the two different sources (CB and PB). When comparing naive and memory conventional T cells (Tcons), it has been noted in murine studies that naive Tcons are the main source of alloreactive cells (51). This is likely the result of restriction of the memory Tcon TCR repertoire to environmental antigens reducing the chance of recognition of minor histocompatibility antigens (51). Whilst the exact specificity of Tregs is unknown, the TCR repertoire, in mice, has been shown to be diverse (52). Consequently, it is possible that CB Tregs are more alloreactive than PB Tregs. Polyclonal expanded CB have demonstrated more suppressive capacity than PB expanded Tregs, in mixed lymphocyte reaction assays, possibly reflecting expansion of a broader alloreactive repertoire (53). The important question then would be, “does a larger pool of alloreactive Tregs in CB translate into a lower dose requirement for cell therapy for graft rejection than with PB Treg sources”? Answering this question will probably require human clinical trials.

### Isolation of Tregs

Several surface cell markers are commonly used to isolate Tregs with high purity from both PB and CB. Tregs from either source have a CD4^+^CD25^hi^CD127^low^ phenotype and, therefore, the most common isolation strategies select for cells with CD4^+^CD25^high^ expression over CD25^low/intermediate^ expression found on T effector cells, with or without additional selection of CD127^low^ expression. Several published Treg isolation protocols also have an additional selection for CD45RA^+^ cells to isolate the naive Treg population (54, 55). In adult PB, it has been proposed that selecting this CD45RA^+^ population leads to a more stable Treg population if the cells are to be repeatedly restimulated during expansion (56).

Currently, clinical grade Treg isolation remains a compromise between what is desirable and what is possible under GMP. Of the groups detailed in Table 1, the majority use magnetic based sorting, as these are closed systems, most of the reagents are CE-certified and validated protocols are available. Many protocols use the CliniMACS system, isolating cells with magnetic beads bound to anti-CD25 antibody. However, optimal purification is difficult as a proportion of Tcons express CD25 to high levels, such that it is difficult to select the CD25^hi^ Treg population alone. An additional clinical grade negative selection for CD4^+^ cells, such as that used to generate research grade Tregs, would require large combinations of antibodies which is impractical in the clinical setting, as each antibody must be GMP validated. Consequently, the majority of studies using the CliniMACS system use an established two-step procedure of CD19/CD8 depletion followed by CD25^+^ enrichment (57–59). However, there are significant disadvantages with this methodology. First, variations in the source of the cells can lead to additional contaminants; Patel et al. found that the standard two-step method produced very poor purity (<10%) when attempting to isolate Tregs from G-CSF mobilized PB (60). Additional CD14 depletion was required to improve purity. Second, even after depleting CD4^−^ contaminants, enrichment with the anti-CD25 clinical reagent leaves significant numbers of CD127^hi^CD25^intermedate^ cells. Even when selecting from more conventional apheresis samples, Treg purities are only between 40 and 60% (57–59, 61) of either CD45^+^ or total events. This led Peters et al. to employ an extra CD127 depletion step in order to achieve a ~90% Treg purity (61); as yet, though, there is no clinical grade anti-CD127 reagent.

**Table 1:** Treg use or planned to be used in clinical trials.

Disease application	Center	Ph	Cell dose	Product	Indication	Effects	Study ID	Ref.
HCT	Gdansk	I	1 × 10^5^–3 × 10^6^/kg	Expanded poly-Tregs	GvHD treatment	Safe/reduced immunosuppression	NKEBN/458-310/2008 (Gdansk ethics board)	(62)
	Minnesota	I	1–30 × 10^5^/kg^a^	Expanded CB poly-Tregs	GvHD prophylaxis	Safe reduced acute GvHD, increased infection	NCT00602693^b^	(44, 63)
	Minnesota	I	3–100 × 10^6^/kg	Expanded CB poly-Tregs with engineered cell line	GvHD prophylaxis	Safe reduced GVHD and no increased relapse	NCT00602693	(45)
	Perugia	I	2–4 × 10^6^/kg	Fresh polyTregs	GvHD prophylaxis	Safe/reduced leukemia relapses/reduced incidence of GvHD	Protocol No 01/08, CEAS Umbria	(64, 65)
	Regensburg	I	≤ 5 × 10^6^/kg	Fresh polyTregs	GvHD prophylaxis	Safe	Treg002EudraCT: 2012-002685-12^c^	(56)
	Milan	I	1–3 × 10^5^/kg	Tr1 (*IL-10 DLI* or *DC-10 DLI*)	GvHD prophylaxis	Safe/long-term disease-free survival in 4 patients	ALT-TEN, IS/11/6172/8309/8391	(66)
	Stanford	I/II	0.1–10 × 10^6^/kg	Fresh polyTregs	GvHD prophylaxis	Terminated (NCT01050764)Recruiting (NCT01660607)	NCT01050764/NCT01660607	–
	Dresden	I	0.6–5 × 10^6^/kg	Expanded polyTregs	GvHD treatment	Tumors in 2 patients/stable chronic GvHD	Protocol no. EK 206082008	(58)
	Bologna	I/II	0.5–2 × 10^6^/kg	Fresh polyTregs	Chronic GvHD prophylaxis	Recruiting	NCT02749084	–
	Minnesota	I/II		Fresh CB polyTregs with IL-2	GvHD prophylaxis	Recruiting	NCT02991898	–
	Boston	I		Fresh polyTregs with IL-2	Steroid refractory chronic GvHD treatment	Recruiting	NCT01937468	–
	Lisbon	I/II	0.5–3 × 10^6^/kg	Fresh polyTregs	Steroid refractory chronic GvHD treatment	Recruiting	NCT02385019	–
	Stanford	I		polyTregs	Steroid-dependent/refractory chronic GvHD treatment	Unknown	NCT01911039	–
	Liege	I	0.5 × 10^6^/kg	Fresh polyTregs	chronic GvHD treatment	Unknown	NCT01903473	–
	Houston	I/II	1–10 × 10^6^/kg	Fucosylated polyTregs	GvHD prophylaxis	Active, not recruiting	NCT02423915	–
	Tampa	I		Donor expanded Tregs	GvHD prophylaxis	Recruiting	NCT01795573	–
	Minnesota	I	3 × 10–100^6^/kg	Induced Tregs	GvHD prophylaxis	Active, not recruiting	NCT01634217	–

Organ trans	London, Oxford,	I/II	1–10^6^/kg	Expanded polyTregs	Living donor kidney transplant	Recruiting	NCT02129881	(67)
	Berlin	I/II	0.5–3 × 10^6^/kg	Expanded polyTregs	Living donor kidney transplant	Recruiting	NCT02371434	(67)
	San Francisco	I/II	4–10 × 10^6^/kg^d^	Donor-alloantigen-reactive Tregs	Living donor kidney transplant	Recruiting	NCT02244801	(67)
	Boston	I/II		Belatacept-conditioned Tregs	Living donor kidney transplant	Active, not recruiting	NCT02091232	(67)
	Chicago	I		Expanded polyTregs	Living donor kidney transplant	Active, not recruiting	NCT02145325	–
	Milan	I/II		Antigen-specific Tr1 (T10 cells)	Living donor kidney transplant	Not yet recruiting		(67)
	Moscow	I	3 × 10^6^/kg^d^	Expanded polyTregs	Kidney transplantation	Unknown	NCT01446484	–
	Multicenter USA	I/II	6 × 10^6^/kg	Donor reactive and polyTregs	Kidney transplantation	Recruiting	NCT02711826	–
	London	I	≤ 4.5 × 10^6^/kg	Expanded polyTregs	Liver transplant	Recruiting	ThRIL, NCT02166177	(68)
	Nanjing	I	1 × 10^6^/kg	Alloantigen-specific Tregs	Liver transplant	Unknown	NCT01624077	–
	San Francisco	I	7 × 10^5^–10 × 10^6^/kg^d^	Donor-alloantigen-reactive Tregs	Liver transplant	Recruiting	NCT02188719	–

Other Treg-based trials	San Francisco	I	5 × 10^6^/kg^d^	Expanded polyTregs	Subclinical rejection in kidney transplantation	Active, not recruiting	NCT02088931	–
	San Francisco	I	4–7 × 10^6^/kg^d^	Donor-alloantigen-reactive Tregs	CNI reduction in liver transplantation	Recruiting	NCT02474199	–

Autoimmunity	Gdansk	I	≤ 30 × 10^6^/kg	Expanded polytTregs	Recent T1D	Safe/reduced insulin doses	ISRCTN06128462^e^	(69–71)
	San Francisco	I	7 × 10^4^–40 × 10^6^/kg^d^	Expanded polyTregs	T1D	Safe	NCT01210664	(72)
	Lille	I/II	1 × 10^4^–10 × 10^6^/kg	Ovalbumin-specific Tr1	Refractory Crohn’s disease	Safe/clinical response in 40% of patients	CATS1	(73)
	Gdansk	I		Expanded polyTregs	Multiple sclerosis	Recruiting		–
	Nanjing	I/II	10–20 × 10^6^/kg	Expanded polyTregs	Autoimmune Hepatitis	Not yet recruiting	NCT02704338	–
	Gdansk	II		Expanded polyTregs	Recent T1D	Recruiting		–
	Hunan	I/II	1–5 × 10^6^/kg	Expanded third-party CB polyTregs	Recent T1D	Recruiting	NCT02932826/NCT03011021	–
	Multicenter USA	II		Expanded polyTregs	Recent T1D	Not yet recruiting	NCT02691247	–
	San Francisco	I	3–20 × 10^6^/kg	Expanded polyTregs	Recent T1D	Recruiting	NCT02772679	–
	San Francisco	I	1.4–23 × 10^6^/kg^d^	Expanded polyTregs	Systemic Lupus erythematosus	Not yet recruiting	NCT02428309	–

*^a^Two infusions day +4 and day +15 after HCT*.

*^b^clinicaltrials.gov*.

*^c^www.clinicaltrialsregister.eu*.

*^d^Converted to cells/kg based on 70 kg average body mass if not stated by study (European standard)*.

*^e^www.isrctn.com*.

As an alternative to CliniMACS selection, our group has been investigating the feasibility of using streptamer technologies to isolate Tregs as part of a European Union funded grant, T-Control. Streptamer based technologies involve a streptactin core conjugated to a magnetic bead and the Fab of an antibody of interest can be reversibly loaded onto the streptactin to create a selecting streptamer. The advantage with this system is that following selection the addition of d-biotin competes with the Fab for the streptacin, dissociating the Fab from the streptamer. The Fabs are designed to be low affinity so, in turn, dissociate from the selected cells leaving and “untouched” cell. This means that it is possible to perform multiple positive selections. It is also possible to select from whole blood without substantial cell processing. This allows for the selection from cryopreserved cell sources such as cryopreserved CB. In our own hands, we have developed a CD4^+^ selection followed by CD25^+^ selection from frozen CB units, to a point where full GMP compliance is possible. It is also possible to select CD4^+^CD25^+^CD45RA^+^ populations using streptamers from adult PB (55).

In view of the poor Treg purities obtained with conventional isolation strategies, a number of groups are now adopting flow cytometry-based Treg purification methods. This allows for purification based on multiple surface markers in one step, including CD127, CD25, CD62L, CD45RA, and CD27 (74). This allows sub-gating to generate a higher purity Treg population or a specific Treg subset (75). However, obtaining clinical grade flow cytometry sorting and reagents represents a major limitation for this technique.

As reviewed by Trzonkowski et al. (74) there are also now a number of new technologies for sorting being developed, such as microfluidic switch technologies (76) and closed cartridge super fast valve sorting (74). Microfluidic switch technologies use a 24-channel sealed microfluidic chip that redirects a fluidic stream using a sealed air bubble system. A prototype sorter was able to select Tregs (CD4^+^CD25^+^CD127^low^ cells) to high purity at very high cell selection rates compared to conventional cell sorters. This is because multiple selections are being performed in parallel, compared to a single droplet stream with conventional flow cytometer sorters (76). Closed cartridge super fast valve sorting (74) uses a magnetic valve on microchip to select the interrogated labeled cells. Both systems have the advantage of using disposable selection chambers (microfluidic or disposable cartridge) and, thus, designed for sterile GMP grade selections from the outset.

### Treg Dose

The optimal Treg dose for each clinical application is crucial because it dictates which Treg source can be used and whether postisolation manipulation and expansion are necessary. If Tregs are to be used unmanipulated, then the potential yield is dependent upon the number of Tregs available in the original source. Using a single unstimulated PB apheresis from healthy donors, Di Ianni et al. were able to achieve an unmanipulated Treg dose of 2–5 × 10^6^/kg (*n* = 21) (64). This Treg dose was used as GvHD prophylaxis, given 4 days prior to HC in the setting of haploidentical HCT. However, the size of the apheresis donation, and hence the Treg dose, is determined by donor characteristics, the PB Treg concentration, and the regulatory and ethical considerations of the host country regarding the apheresis procedure.

Similarly, when using unmanipulated Tregs isolated from CB, the Treg dose is limited by the size of CB units currently banked. The largest CB units banked at the Anthony Nolan Cord Bank are 2.9 × 10^9^ TNC (77). Consequently, with an average CD4 content of 16% (78) and Treg content of 6% of CD4^+^ T cells (47), the maximum predicted Treg yield possible would only be 28 × 10^6^ cells, even with 100% efficiency. Realistically, practical cell yields from frozen UCB are considerably lower than this with Brunstein et al. reporting 0.1–7 × 10^6^ Tregs postisolation (45) and Parmar et al. 0.5–3.0 × 10^6^ cells (79).

Given the low Treg numbers isolated from PB and CB, many groups have focused on developing expansion protocols to achieve larger target doses (expansion methods will be discussed later). In phase I studies, expanded PB Treg lines have been used for prophylaxis and/or treatment of GvHD at ranges between 0.1 and 5 × 10^6^ cells/kg (58, 62). In the setting of autoimmunity, expanded polyclonal PB Tregs have been used at doses as low as 0.06 × 10^6^ cells/kg and as high as 23 × 10^6^ cells/kg (67, 69–71). Expanded CB Tregs have been used in two studies as prophylaxis of GvHD, with doses between 0.1 and 3 × 10^6^ and 3–100 × 10^6^ cells/kg (45, 63).

The majority of Tregs clinical studies performed so far are phase I safety studies (discussed below), demonstrating Tregs appear safe and tolerated over a range of doses. However, can we predict a required Treg dose for clinical efficacy? Tregs suppress a range of immune cell types using a many of different mechanisms in animals and humans (80, 81). Consequently, estimating cell dose is complicated. Tang and Bluestone predicted an effective Treg dose based on the suppression of Tcons (1). This calculation was based upon allometric scaling of the suppression of the resident Tcons pool in animal models. Proof of principle experiments in animal models suggest that a higher Treg to Tcon ratio, amounting to raising the proportion of Tregs of CD4^+^ cells from 5–10 to ~30%, is required to control responses to grafts (82–84). Since the average adult human has ~150 × 10^9^ CD4^+^ T cells, and 13 × 10^9^ Tregs, raising the total Treg pool to 30% would require 53 × 10^9^ Tregs (or ~700 × 10^6^/kg for 70 kg individual) (1). This is untenable based on current Treg isolation and production technology. However, even in phase I studies, efficacy has been observed with lower doses [between 1 × 10^5^ and 4 × 10^6^/kg (62, 64, 66)]. It is notable that these are all in a lymphodepleted context, either as a result of preconditioning for HCT or during immune recovery following HCT (62, 64, 66).

A further complication to estimating the required dose for clinical use is the issue of trafficking and specificity; where do the Tregs need to be to exert their function and what proportion of the Treg pool are responding? These factors are known to impact on allometric scaling calculations (retention in tissues, active proliferation in lymph nodes) (85) and may also impact on the current clinical trials using Tregs (86). Recent trials have shown that increased total Treg dose did not lead to increased Treg presence in the periphery (45, 63). With the application of Treg clinical trials, there has been increased visualization of Tregs *in vivo*, as studies seek to determine efficacy and longevity (72). This should have the added benefit of also indicating where these cells are going and, therefore, what trafficking markers are desirable, i.e., if efficacy correlates with the Tregs being present in a particular tissue, the effective dose might be improved by engineering markers that direct the manufactured cells to that site. Consequently, the “effective” dose may be improved by postmanufacture/expansion modification.

At present, there are only a few studies actively attempting to alter Treg migration with the aim of improving efficacy for Treg therapy. Of particular note are the studies investigating fucosylation of Tregs. In 2005, using murine studies, it was shown that knocking out a key step in the generation of selectin ligands (fucosylation of glycoproteins) prevented the expression of E and P-selectins without affecting the expression of other homing receptors (CD62L, β_1_-integrin, LFA-1) or suppressive function. However, it prevented the cells from migrating to the footpad of mice in a skin inflammation model, and subsequently blocked their ability to suppress *in vivo* inflammation (87). This, in turn, lead to a proof of principle study using a humanized murine model system with NSG mice being injected with human PBMC to generate a GvHD effect. *Ex vivo* fucosylated CB Tregs demonstrated higher levels of binding E-selectin, were more potent suppressors of GvHD and persisted longer than untreated CB Tregs (88). Studies of autoimmunity also suggest a role for CCR2 in Treg migration; in collagen induced arthritis animal models, blockade of CCR2 could prevent initiation of arthritis, but once established, exacerbated it by interfering with the function of CCR2^+^ Tregs. For other potential homing markers perhaps the best source would be those implicated in recruiting Tregs as a part of tumor immune evasion. These include CXCR3, CCR6, CCR5, CXCR4, CCCR8, and CCR10, as they have all been implicated in recruiting Tregs to tumor sites (89), as reviewed by Adeegbe and Nishikawa (90).

### Treg Specificity

**Figure 1:**
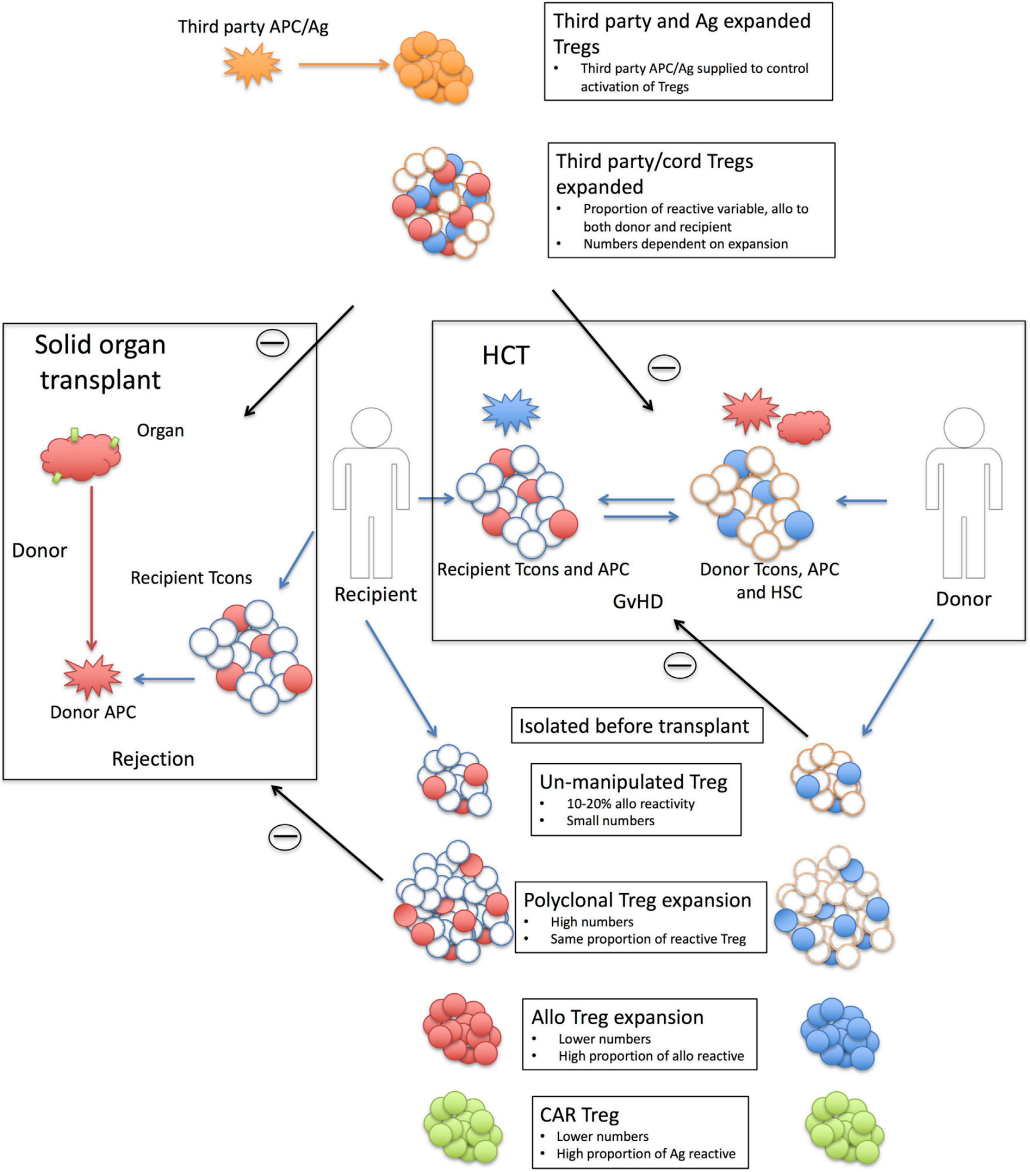
Strategy behind Treg therapies in solid organ and hematopoietic cell transplantation. Shown are the strategies associated with using Tregs in solid organ (left hand side) and hematopoietic cell transplantation (HCT) (right-hand side). Solid organ transplant; transplanted tissue [red HLA antigen (Ag) with green tissue antigens] are rejected by recipient conventional T cells (Tcons) recognizing donor APC/Ag (red). Only a proportion of the donor T cells will react to the donor antigen-presenting cells (APCs) (10–20% alloreactive T cells shaded in red). Recipient or third-party Tregs are isolated pretransplant with six strategy types; Unmanipulated Tregs are small in number and only a small proportion will be allo-reactive (shaded in red). This proportion is unknown but is likely to be in the same order as the proportion of alloreactive Tcons. Polyclonal expanded Tregs are larger in numbers but the same proportion of alloreactive Tregs (red shaded). Alloexpanded Tregs are expanded to donor APCs and while the resulting product will have a smaller cell number than polyclonal expansion there will be a higher proportion of donor reactive Tregs (red shaded). Chimeric antigen receptor (CAR) Tregs are recipient Tregs modified to recognize tissue antigen (green). Third-party Tregs, expanded are polyclonal expanded third-party Tregs that rely on the alloreactivity of the Treg population. As these are third party, this proportion maybe different to the recipient Treg populations and they may react to both donor and recipient (red and blue shaded Tregs). Third-party and Ag expanded Tregs are third-party Tregs (adult or cord) expanded to a third-party antigen (orange) not present in either the recipient or the donor. The third-party antigen can be supplied (either as APC or antigen) to the recipient and suppression of the rejection event is through bystander suppression. Withdrawing the antigen should then reduce the activation of the Tregs. HCT; Recipient (R) T cells (red shaded) respond to in coming donor (D) APCs (red) for a recipient vs. donor graft-versus-host disease (GvHD). Donor T cells (blue shaded) react to recipient APCs (blue) for a donor vs. recipient GvHD reaction. The levels of recipient cells will depend on the level of preconditioning (MAC or RIC) and the amount of mismatch. Treg therapy; Donor or third-party Treg are harvested. Unmanipulated Tregs; as with solid organ but target is now recipient antigens. Polyclonal expanded Tregs; as solid organ. Alloexpanded Tregs; donor Tregs expanded to recipient APCs. CAR Tregs; as solid organ but unique recipient Ag third-party allo Tregs; as with solid organ. Third-party Tregs expanded: as with solid organ.

To date, Treg dose requirements have been based on therapies using polyclonal Tregs (see below). However, evidence from animal models suggest that graft tolerized animals generate a Treg population that can confer protection (91, 92) and are more potent than Tregs from non-tolerized animals (93, 94). Part of this tolerance mechanism is due to an increased proportion of alloantigen-specific Tregs (91–94) (Figure 1). Murine and human *in vitro*, experiments to expand alloantigen-specific Tregs also demonstrated that they are substantially more potent than polyclonal expanded Tregs (95–98). Consequently, this has led to a number of trials using GMP compliant donor expanded allogenic-specific Tregs (see below), mostly in a solid organ background and part of the ONE study (67). It is hoped that these will be more efficacious than polyclonal expanded Tregs and thereby will require a lower dose. In the kidney and liver transplant settings detailed in the ONE study, recipient Tregs were exposed to donor B cells, i.e., the direct pathway of allorecognition (67). However, a major arm of tissue rejection is through the indirect pathway, namely, donor alloantigens presented to recipient T cells by recipient APCs. Unfortunately, expanding alloantigen-specific Tregs through this pathway has proven more challenging due to the low frequency in the periphery (99).

An alternative is to produce a specific Treg population to a known antigen (Figure 1). This has the advantage of reducing off-target suppression (as only the cognate antigen will activate the Tregs); bystander suppression is still likely to occur, but by controlling the availability and delivery of the antigen, a level of both antigen and tissue specificity could be imposed. As proof of principle, in animal models, Tregs raised to an exogenous antigen (HY peptide in female B6 mice) can prevent the induction of GvHD by allogenic BM and T cells [B6 BM and T cells into C57BL/6 (B6) X (C3H) F1 females] when the antigen is provided (either HY pulsed DCs or HY peptide) (100). Similarly, this could be an induced Treg population, as in the case of ovalubumin-specific induced type-1 Tregs (Tr1) [cells being trialed in Crohn’s disease (73)]. Finally, specificity could be conferred onto a polyclonal Treg population for a particular application; for example, in the case of therapeutic protein replacement, such as factor VIII in hemophilia treatment, undesirable immune responses to the protein can be prevented using Tregs transduced with a factor VIII-specific TCR (101). Perhaps, however, of greatest interest is the application of chimeric antigen receptor (CAR) technology (99) (Figure 1). CARs, an extracellular antigen-binding domain linked to a intracellular TCR and costimulatory domain, are now being used to treat Leukemia/lymphomas (102) by conferring antigen specificity onto a polyclonal T cell population. If an antigen is known, this can also be applied to a Treg population. Proof of principle has been demonstrated by Elinav et al. with a colitis-specific antigen (103) and Fransson et al. with myelin oligodendrocyte glycoprotein-specific CAR Tregs to protect against experimental autoimmune encephalomyelitis, a model of multiple sclerosis (104).

### Polyclonal Treg Expansion

The majority of published Treg expansion protocols use polyclonal expansion of Tregs, aiming to maintain the thymically derived, natural Treg or, as recently adopted, thymic Treg (tTreg) phenotype (105). Multiple studies have now reported expanding tTregs from both PB or CB, and expansion conditions have become increasingly well defined and translated into GMP compliant protocols (62, 63, 106). Most protocols use anti-CD3 antibody attached to beads, in combination with anti-CD28 costimulation and IL-2 (range, 300–1,000 IU/ml) (62, 106, 107). Using these methods, average expansions of 500–600-fold have been achieved with PB Tregs (62, 70, 72). When expanding PB Tregs, Rapamycin [~100 nM (107–109), murine and human studies] is often added to expansion cultures, sometimes in combination with Retinoic acid [10 µM in serum; 10 nM in serum free conditions (108)]. This is to prevent the outgrowth of contaminant effector T cells and to promote Treg expansion, especially in the case of multiple restimulations (107). In the presence of Rapamycin, 100–1,000-fold expansions have been achieved (with a single restimulation) (107).

When using CB Tregs, anti-CD3/28 bead based methods have produced 100–1,000-fold expansion (110). Brunstein et al. (2016) have, however, been able to achieve much greater expansion from CB Tregs using an engineered cell line as the expanding stimulus (10,000-fold expansion in 2 weeks) (45). Their protocol used anti-CD3 antibody-loaded K562 cells modified to express the high affinity Fc receptor (CD64) and CD86 the ligand of the costimulatory receptor CD28 (45).

### Alloantigen-Reactive Tregs Expansion

It is estimated that the frequency of direct allo-reactive Tregs in adult PB is around 1–10% of Tregs (95, 111). The alloreactive population in CB may be higher as they are naive, but this is difficult to determine as yet there have been no published large-scale expansions to alloantigens with CB. Allo-reactive Tregs can be expanded toward donor APC such as DCs, B cells, and PBMC (41, 95–97, 112). As part of the ONE study to prevent kidney and liver transplant rejection, Putnam et al., developed a Treg expansion method using CD40L activated B cells from the donor to expand recipient Tregs (113) (Table 1). Following the primary stimulus, the expansion is continued with anti-CD3/28 beads to give a 200–4,000-fold expansion after 16 days. Attempts to generate indirect stimulated alloreactive Tregs (alloantigen presented by self APC) have been less successful (95, 114) due to a 100-fold lower frequency of indirect alloresponsive cells (95). Therefore, to generate an alloantigen-specific population the use of transgenic TCR Tregs may be a better, alternative strategy (115).

In both polyclonal and alloantigen driven expansion, it might be possible to introduce traceable markers, chemotactic receptors or drug-inducible suicide genes. These designer features would allow monitoring and control of trafficking as well as the ability to switch off the expanded cells if any adverse reactions were detected (1).

### Assessing Phenotype and Function after Expansion

Following expansion, Tregs should retain their tTreg phenotype as defined by CD4^+^CD127^low^CD25^+^FOXP3^+^CD62L^hi^CCR7^+^ T cells (45). Although FOXP3 expression is vital for Treg function, FOXP3 can also be expressed on activated Tcons and so, in itself, does not distinguish between activated Tregs and Tcons (116). However, FOXP3 expression should be high and sustained compared with Tcons (117); a comparison with expanded Tcons line would be required to assess this. Expanded Tregs should also retain a central memory phenotype characterized by CD62L and CCR7^hi^ expression (45); CD62L expression has been linked to a more suppressive population following adult PB Treg expansion (106) and the expression of CD62L and CCR7 are predictive of *in vivo* function as they allow trafficking to lymphoid tissues in murine models (118). In addition, Helios expression (Ikaros transcription family), has been associated with tTregs (119), and its presence in expanded cells is an additional indication that the cells have retained a tTreg phenotype (45).

Expanded cells should be able to suppress; with suppression classically being defined as inhibition of *in vitro* target adult PB T cell proliferation in coculture assays. Proliferation can be measured be either [^3^H] thymidine ([^3^H]tydr) incorporation (8) or CFSE dilution by flow cytometry (120). However, there are limitations to both techniques, as discussed extensively by McMurchy and Levings (121). These essentially concern false positives and negatives for suppression since all proliferating cells will uptake [^3^H]tydr, the assay relies on the premise that Tregs are hypo responsive, such that only target Tcons proliferation is measured (122). However, Tregs are not hypo responsive murine *in vivo* models (123), and so failure to detect inhibition of proliferation would not refute suppressive function. Labeling the target CD4^+^ cells with CFSE does overcome this limitation as it means that proliferation can be monitored by flow cytometry and any proliferating Tregs excluded (120). Both methods can falsely assign suppressive function to very active Tcons (such as a Tcons cell line) (121), though very active Tcons, that are releasing IL-2, can cause the peak proliferation in the coculture to occur earlier than with Tcons only. This can result in the [^3^H]tydr being added too late for peak proliferation (usually added in the last 12–16 h of culture) while in the case of CFSE this can lead to exhaustion and or cytotoxicity. Both of these states would result in inhibition of proliferation (121). The use of non-Treg controls can illustrate the extent of the issue and an alternative is to measure the inhibition of proinflammatory cytokine release (INFγ and TNFα) from the target cells (124). However, even if suppressive function is demonstrated this does not conclusively prove that tTregs have been expanded over induced Tregs. Thus, a better confirmation seems to be the stability of the FOXP3 expression. The methylation state of the FOXP3 locus is indicative of recent chromosomal remodeling and thus distinguishes between FOXP3 induction over constitutive expression. The study of Treg development in the thymus indicates that the FOXP3 promoter becomes demethylated during development and indicates a stable commitment to the Treg lineage in mice and humans (125–128).

### Clinical Application of Tregs—The Experience to Date

There are an increasing number of clinical trials looking at the safety of Tregs as cellular therapy as reviewed by Trzonkowski et al. and Gliwiński et al. (74, 129) and updated in Table 1. To date, there have been seven trials performed in the HCT setting (Table 1) and nine planned or in progress in solid organ transplantation. There are also at least five phase I clinical trials in autoimmunity, notably type I diabetes, with three transitioning from phase I to phase II studies (Table 1).

In Minnesota, Brunstein et al. performed two phase I clinical studies of third-party CB Tregs in the setting of double UCB transplantation. In the first study (2011), 23 adults (median 52 years, range 24–68) were infused with *ex vivo* isolated and polyclonal expanded (anti-CD3/28 beads) CB Tregs, at doses between 0.1 and 3 × 10^6^ cells/kg (63). In this dose escalation study, Tregs were infused on day +1 and +15 and patients also received standard GvHD prophylaxis (Ciclosporin or Mycophenolate/Sirolimus). Treg infusions were well tolerated with no infusion toxicities reported, although, they did note an increase in viral reactivation compared with historical controls (44). More recently, 11 patients (median age 61 years, range 45–68) receiving an umbilical CB transplant, were given Treg doses of 3–100 × 10^6^ cells/kg, expanded using the transgenic K562 cell line (45). Again, these patients received standard GvHD prophylaxis (Sirolimus and Mycophenolate). In this study, there were no adverse events reported and the incidence of acute GvHD was low compared to 22 historical controls.

In 2011, Edinger et al. reported a small phase I clinical trial using PB isolated Tregs postallogeneic HCT. After cessation of GvHD prophylaxis (within 1 year), nine patients at high risk of disease relapse were given up to 5 × 10^6^ Tregs/kg of unmanipulated magnetic bead sorted PB Tregs. This was followed by a donor lymphocyte infusion (DLI) 8 weeks later. While this was only a small study, there were no adverse reactions, relapse or GvHD (56).

Perhaps more ambitiously, Di Ianni et al. in Perugia have performed a larger study in haploidentical HCT with unmanipulated donor PB Tregs being administered (day −4) prior to HSC (9.4 × 10^6^/kg) and escalating dose of Tcons (day 0). In this study, the Treg infusion was the only GvHD prophylaxis used. Twenty-eight patients were given a fixed dose of 2 × 10^6^/kg magnetically separated Tregs and a dose escalation of Tcons from 0.5 × 10^6^, 1.0 × 10^6^, 2.0 × 10^6^ to 4.0 × 10^6^/kg; the majority of patients (*n* = 17) received 1 × 10^6^/kg Tcons. Onset of acute GvHD was the indication for stopping the dose escalation with two out of the five patients that received 4 × 10^6^ Tcons/kg developing ≥grade 2 GvHD. Although 50% of patients relapsed, all the patients had high-risk disease and was lower than an equivalent historical control group (64). A similar study is also being performed in Stanford (Table 1).

In Dresden, Theil et al. used polyclonal expanded PB Tregs to treat five patients with steroid refractory chronic GvHD (58). Bead separated, donor derived Tregs were expanded with anti-CD3/28 beads (300–1,000 IU/ml IL-2) and administered at doses of 0.6–5 × 10^6^/kg. Three of these patients also received low dose IL-2 (0.3 × 10^6^ IU/m^2^/day) to promote *in vivo* Treg expansion. Interestingly, there was an increase in Treg numbers in the IL-2-treated group but these seemed to be of endogenous origin (naive phenotype). Two patients had improved symptoms and three patients were able to reduce immunosuppression. Two patients developed skin tumors, which may have been exacerbated by the Tregs. However, as noted by the authors of the study, all patients received significant other immunosuppressive therapy, including transplant conditioning with Fludarabine, Methotrexate, total body irradiation, anti-GvHD therapy of tacrolimus, mycophenolatemofetil, extracorporeal photopheresis and, during Treg therapy, steroids such as Prednisolone. Secondary tumors are increased postallogeneic HCT and with such profound general immunosuppression (130).

Although several trials in solid organ transplantation are currently in progress (Table 1), the only other field where Treg clinical trials have been reported is autoimmunity. In particular, there are two studies on the use of Tregs in type 1 diabetes (TD1), one in pediatric and one in adult patients. The use of expanded autologous Tregs to treat early TD1 was pioneered in Poland (69–71). Using flow sorted polyclonal expanded Tregs, in a succession of studies, Marek-Trzonkowska et al. have now treated 12 children. The idea was to use expanded Tregs to boost the protection of the beta-islet cells in the pancreas against autoimmune attack in the early stages of TD1. Most of the patients were between 8 and 16 years and within 2 months of diagnosis. Treg doses were between 10 and 30 × 10^6^/kg and were more practical to achieve than with adult patients. After one year, no serious adverse events were reported and 8 out of 12 patients continued to be in clinical remission. Two remained insulin independent. Overall, insulin doses continued to be significantly lower than in an untreated control group suggesting that beta cells functionality had been protected to varying degrees (71).

The results of a similar study in San Francisco with adult TD1 patients is perhaps less encouraging; 14 patients (mean age 30, mean time after diagnosis 39 weeks) were treated in four cohorts with escalating doses of 0.06 × 10^8^, 0.4 × 10^8^, 4 × 10^8^, and 23–27 × 10^8^ (fixed doses rather than body weight) of flow sorted, polyclonal expanded Tregs. Whilst there were no adverse events, they only observed modest effects on insulin production with a decline in some cases, although this did not correlate with Treg dose. Of note, they were able to detect the transplanted Tregs up to 90 days after infusion by utilizing deuterium labeling of the cells during the expansion (72).

In summary, in HCT the Tregs have been well tolerated, although with mixed data toward viral reactivations with CB and skin tumors with PB Tregs. Efficacy has been more difficult to determine, as most of the reported studies are phase I safety trials. Being safety studies, the immunosuppression was often still present and these agents will also affect the Tregs, further complicating any indications of efficacy. However, a number of phase II studies are now planned or in progress (Table 1) and should progress our understanding further.

## Concluding Remarks

Regulatory T cells are able to suppress the function of many cells types using a variety of cell contact dependent and independent mechanisms. In theory, therefore, Tregs therapies could potentially cause general immunosuppression, much like standard immunosuppressive drugs they may replace. However, early *in vitro* animal models in HCT demonstrated the Tregs have the potential to suppress GvHD while maintaining a GVL effect (38). Furthermore, over the last few years, there have been an increasing number of phase I clinical studies reported, demonstrating the safety of Tregs cellular therapies. In the clinical trials to date, even those using high Tregs doses (100–500 × 10^6^ cells/kg), there has been no conclusive evidence of general immunosuppression, in terms of increased relapse after HCT, higher numbers of opportunistic infections or outgrowth of tumors. As the field of Treg cellular therapy advances, it is now hoped that the results of the phase II/III clinical Treg studies in progress will answer the question of whether Tregs have efficacy *in vivo* in preventing allo and autoimmune complications.

Clearly, however, a number of important hurdles remain; what is the best cell source, how can Tregs be isolated practically and safely, and what Treg cell dose should be given? Added to this are aspects of specificity; can a more directed Treg population allow for a lower dose to be administered and, which antigens should they be directed toward? The next round of clinical studies, especially the ONE study, will hopefully answer some of the questions of efficacy, although these are mainly in the field of solid organ transplant. Also, whichever method of isolation and/or expansion is employed, the cost of producing these cells for clinical use can be prohibitive. Therefore, if Treg therapies are to become viable, multicenter collaboration, and large phase III randomized trials will be required and are likely to require the assistance of commercial pharmaceutical companies. As with many biological cell-based therapies though, they have the potential to be more effective than pharmacological therapies.

## Author Contributions

RD, RD, AM, and AS all contributed to writing the manuscript.

## Conflict of Interest Statement

AS is now an employee of GlaxoSmithKline. All other authors declare no conflict of interest.
